# Boosting scRNA-seq data clustering by cluster-aware feature weighting

**DOI:** 10.1186/s12859-021-04033-7

**Published:** 2021-06-02

**Authors:** Rui-Yi Li, Jihong Guan, Shuigeng Zhou

**Affiliations:** 1 Department of Computer Science and Technology, Tongji University, 4800 Caoan Road, Shanghai, 201804 China; 2 Shanghai Key Lab of Intelligent Information Processing, and School of Computer Science, Fudan University, 220 Handan Road, Shanghai, 200433 China

**Keywords:** Single cell RNA sequencing, Feature weighting, feature selection, Clustering

## Abstract

**Background:**

The rapid development of single-cell RNA sequencing (scRNA-seq) enables the exploration of cell heterogeneity, which is usually done by scRNA-seq data clustering. The essence of scRNA-seq data clustering is to group cells by measuring the similarities among genes/transcripts of cells. And the selection of features for cell similarity evaluation is of great importance, which will significantly impact clustering effectiveness and efficiency.

**Results:**

In this paper, we propose a novel method called CaFew to select genes based on cluster-aware feature weighting. By optimizing the clustering objective function, CaFew obtains a feature weight matrix, which is further used for feature selection. The genes have large weights in at least one cluster or the genes whose weights vary greatly in different clusters are selected. Experiments on 8 real scRNA-seq datasets show that CaFew can obviously improve the clustering performance of existing scRNA-seq data clustering methods. Particularly, the combination of CaFew with SC3 achieves the state-of-art performance. Furthermore, CaFew also benefits the visualization of scRNA-seq data.

**Conclusion:**

CaFew is an effective scRNA-seq data clustering method due to its gene selection mechanism based on cluster-aware feature weighting, and it is a useful tool for scRNA-seq data analysis.

## Background

Single cell RNA sequencing (scRNA-seq) is a novel technology to uncover the heterogeneity of cells, which can overcome the limitations of traditional RNA sequencing technologies in detecting slight expression difference among cells [1–3]. Major scRNA-seq data analysis tasks include de-noising [4], batch effect elimination [5], clustering analysis [6] and visualization [7]. Among them, clustering analysis is particularly important for studying cell heterogeneity. The purpose of scRNA-seq data clustering is to partition a set of cells into a certain number of homogeneous groups, each of which is referred to as a cluster. Clustering has been applied to solving many biological problems such as detecting new cell types [8], cell lineage tracking [9–11], studying pathogenic mechanism [12], exploring drug responses and even disease diagnosis [13, 14].

Up to now, a number of clustering methods have been proposed specifically for scRNA-seq data, which exploit the unique characteristics of scRNA-seq data, including high sparsity, dimensionality and noise that seriously challenge the conventional clustering algorithms [15–17]. Recently, Li, Guan and Zhou conducted a comprehensive survey and comparison study on scRNA-seq data clustering algorithms [18], where existing scRNA-seq data clustering methods are classified into six types: *distance-based*, *density-based*, *graph-based*, *matrix-based*, *model-based* and *deep learning-based*.

Distance-based methods use distance-based clustering algorithms, such as *k*-means and hierarchical clustering. The majority of scRNA-seq clustering methods in the literature such as SC3 [19], SINCERA [20], CIDR [21], RaceID [22] and pcaReduce [23] fall into this category. Out of which, SC3 is a consensus clustering method that applies three kinds of similarity measurements and two kinds of feature transformation techniques to integrate clustering results [19]. SINCERA is a package for scRNA-seq data analysis, where some pre-process steps such as normalization and quality control are performed before clustering, and hierarchical clustering is applied to the similarity matrix generated by centered Pearson’s correlation and average linkage [20]. CIDR performs hierarchical clustering on a few top principal coordinates obtained by principal coordinate analysis (PCoA) over the Euclidean distance matrix of samples [21]. pcaReduce is an agglomerative clustering approach that integrates principal components analysis and hierarchical clustering [23].

Density-based methods employ density-based clustering mechanisms such as the DBSCAN algorithm [24] and its variants [25]. For example, Jiang et al. [26] proposed a method named giniClust to identify rare cell types in scRNA-seq data. They applied the DBSCAN algorithm after selecting significantly different genes based on the Gini index. Graph-based methods first transform the data to a graph, over which a graph clustering algorithm is applied. Two examples of this type are SNNCliq [27] and Seurat [28], both discover sub-graphs on the Shared Nearest Neighbors (SNN) graph. The probability model-based methods cluster data based on a certain probability distribution or process. For example, DIMM-SC [29] is specifically proposed for processing droplet-based scRNA-seq data, based on the Dirichlet Mixture Model. Prabhakaran et al. [30] proposed another probability model-based method to correct technical variations based on the Dirichlet Process. The matrix-based methods first derive a matrix from the original scRNA-seq data, and then perform matrix splitting or decomposition to cluster data. Representative algorithms include the method based on nonnegative matrix factorization (NMF) [31] and the BackSPIN [32] based on sorting points into neighborhoods (SPIN).

With the growth of scRNA-seq data, deep learning (DL) is applicable to scRNA-seq data clustering. For example, Eraslan et al. [4] proposed a deep count auto-encoder network (DCA) to de-noise scRNA-seq data and then cluster the data with the features extracted by a multi-layer neural network. Lopez et al. [33] introduced the scVI method to derive probabilistic representations of scRNA-seq data from deep generative model of variational auto-encoder. DL-based scRNA-seq data clustering methods try to learn representations of scRNA-seq data through deep neural networks. In essence, they are owned by a kind of nonlinear feature transformation techniques.

Actually, most existing methods exploit the similarity between cells to do scRNA-seq data clustering, while similarity evaluation relies on the selection of genes. Consequently, how many and which genes are used for clustering is particularly important. Currently, there are mainly two types of gene-filtering strategies: (1) *threshold-based* approaches that select genes whose expression values satisfy a certain threshold, and (2) *variation index-based* approaches that measure the variation of gene expression values in different cells and then select genes with large variations.

For instance, SC3 uses a threshold-based approach to filter genes before clustering. Specifically, it removes genes/transcripts that are either expressed (expression value > 2) in less than *X*% of cells (rare genes/transcripts) or expressed (expression value > 0) in at least $(100-X)$% of cells (ubiquitous genes/transcripts), where the default value of *X* is 6 [19]. Some additional methods like NMF-based clustering methods and RaceID also remove genes of low expressions [22, 31]. As for the second type, the GiniClust algorithm selects high Gini-index genes by fitting the relationship between the Gini index and max gene expression level with LOESS regression [26]. BackSPIN selects the top 5000 genes as informative features based on *coefficient of variation* (CV), defined as the standard deviation divided by the mean [32]. Generally, threshold-based approaches can be regarded as a kind of simplified denoising methods, while variation index-based methods select differentially expressed genes. However, neither of them takes the goal of clustering into account.

In this paper, we propose a clustering-aware feature weighting method **CaFew** for scRNA-seq data clustering. First, by resolving the optimization problem of clustering, a weight matrix indicating the importance of features in different clusters is derived. Then, we select genes based on the weight matrix. Concretely, we select those genes with a relatively large weight in at least one cluster or a large weight variation across different clusters. Experiments over several benchmark datasets show that CaFew can effectively boost clustering performance. What is more, by combining CaFew with SC3 (denoted as “CaFew+SC3”), we achieve the state of the art performance. Finally, CaFew is also helpful for scRNA-seq data visualization.

## Results

In this section, we evaluate CaFew in clustering scRNA-seq data. First, we introduce 8 publicly available scRNA-seq datasets and clustering evaluation metric. Then, we present the experimental results of CaFew on these datasets, including selected features, clustering accuracy and visualization.

### Datasets and performance metric

We collect 8 publicly available scRNA-seq datasets with ground-truth cell type information. Table 1 presents the statistical information of these datasets, including the number of cells, clusters and genes and their sequencing protocols. We can note that these datasets range in size from dozens to thousands, with more than 15,000 genes/transcripts. The number of cell types varies from 3 to 16. Units of gene/transcript levels include FPKM (Fragments Per Kilobase of exon model per Million mapped reads), CPM (Counts of exon model per Million mapped reads) and UMI (Unique Molecule Identifier). Specifically, UMI uses a direct measurement of transcript copies for each transcript [34], while the other two metrics normalize the raw read counts based on sequencing depth and gene length. In addition, these scRNA-seq data were generated from some representative sequencing platforms, such as Smart-Seq2 [35], Microwell-seq [36] and 10X [37] etc.

In our experiments, we use Adjusted Rand Index (ARI) to measure the clustering performance. Given the ground truth class assignments $labels\_true$ and the predicted class assignments $labels\_predict$, ARI measures the similarity of these two assignments [38]. Concretely, the overlapping between two assignments can be summarized as a contingency table, which reports the intersection cardinality of each true-predicted cluster pair. ARI is calculated as follows:

1 $$\begin{aligned} ARI= \frac{\sum _{ij}\left( {\begin{array}{c}t_{ij}\\ 2\end{array}}\right) - \left[ \sum _{i}\left( {\begin{array}{c}a_i\\ 2\end{array}}\right) \sum _{j}\left( {\begin{array}{c}b_j\\ 2\end{array}}\right) \right] / \left( {\begin{array}{c}m\\ 2\end{array}}\right) }{\frac{1}{2}\left[ \sum _{i}{}\left( {\begin{array}{c}a_i\\ 2\end{array}}\right) + \sum _{j}{}\left( {\begin{array}{c}b_j\\ 2\end{array}}\right) \right] - \left[ \sum _{i}\left( {\begin{array}{c}a_i\\ 2\end{array}}\right) \sum _{j}\left( {\begin{array}{c}b_j\\ 2\end{array}}\right) \right] / \left( {\begin{array}{c}m\\ 2\end{array}}\right) } \end{aligned}$$

where *m* is the number of cells totally in the dataset, $t_{ij}$ is the value at the *i*th-row and the *j*th-column in the contingency table, $a_i$ is the sum of the *i*th-row of the contingency table, $b_j$ is the sum of the *j*th-column of the contingency table, and () denotes a binomial coefficient. ARI ranges from − 1 to 1, where a negative value means mismatch and ‘1’ indicates a perfect match.

**Table 1:** A summary of 8 sc-RNAseq datasets

Datasets	#Cells	#Clusters	#Genes	Unit	Sequencing protocol
GSE59892 [54]	49	3	25737	FPKM	Smart-seq [55]
GSE36552 [56]	90	7	19595	FPKM	Tang et al [57]
E-MTAB-3321 [58]	124	5	28223	CPM	Smart-Seq2 [35]
GSE51372 [59]	187	7	15584	FPKM	Tang et al [57]
E-MTAB-2600 [60]	704	3	21231	CPM	Smart-Seq2 [35]
GSE108097 [36]	2746	16	20670	UMI	Microwell-seq [36]
GSE60361 [32]	3005	9	19972	UMI	Islam et al.[34]
SRP073767 [37]	4271	8	16449	UMI	10X [37]

### Feature selection results

As there are two screening steps in the CaFew algorithm, we present the number of genes remained after the first and second screening steps respectively as “#Genes-S1” and “#Genes-S2” in Table 2, where we also present the ratio of selected genes over the total genes.

**Table 2:** Feature selection results of the 8 sc-RNAseq datasets

Datasets	#Genes	#Genes-S1	#Genes-S2
GSE59892	25737	9894 (38.4%)	765 (3.0%)
GSE36552	19595	9786 (49.9%)	283 (1.4%)
E-MTAB-3321	28223	9948 (35.2%)	904 (3.2%)
E-MTAB-2600	30768	9897 (32.2%)	981 (3.2%)
GSE51372	29018	3922 (28.5%)	173 (0.7%)
GSE60361	19972	6740 (33.7%)	79 (0.4%)
GSE108097	20670	8814 (42.6%)	921 (4.5%)
SRP073767	16653	8997 (54.0%)	830 (5.0%)

We can notice that more than half of the features are removed after the first filtering step. For example, there are more than 20000 genes in GSE59892. Only 9894 features are retained after the first filtering step, accounting for 38.4% of the total genes. After the secondary screening step, only a few hundred features remain, which is less than 5% of the total genes. In conclusion, CaFew can select much fewer genes that are conductive to clustering.

### Effect of gene selection on clustering

Here, to check whether the selected genes are more effective in exposing the cluster structures in datasets, we apply the Davies–Bouldin index (DBI) to the 8 datasets, which is defined as follows [39]:

2 $$\begin{aligned} DBI=\frac{1}{k}\sum _{i=1}^{k}\min _{j\ne i}\frac{d_i+d_j}{d_{ij}} \end{aligned}$$

where $d_i$ is the average distance between each sample and the centroid of cluster *i*, $d_{ij}$ is the distance between the centroids of clusters *i* and *j*, and *k* is the number of clusters. Obviously, the smaller DBI is, the more compact the clusters.

Table 3 presents the DBI values before and after gene selection on the 8 datasets. We can see that after using CaFew for feature selection, the DBI for all the datasets is significantly lower than that when all features are used. This result indicates that after selecting features based on feature weighting, the cluster structure is clearer, which shows that our feature selection is helpful to cluster.

**Table 3:** DBI values of datasets before and after feature selection

Datasets	Genes-all	Genes-S1	Genes-S2
GSE59892	2.03	2.12	1.81
GSE36552	1.99	2.15	1.51
E-MTAB-3321	3.31	3.20	3.15
E-MTAB-2600	7.80	7.69	7.59
GSE51372	4.48	4.08	2.87
GSE60361	5.95	5.93	3.80
GSE108097	7.98	7.49	5.59
SRP073767	10.85	10.04	6.05

### Clustering performance

To demonstrate the effectiveness of CaFew, we compare the clustering performance before and after it is applied to different clustering methods, including traditional clustering algorithms and several high-performance clustering algorithms proposed specifically for scRNA-seq data.

#### Results of traditional clustering algorithms

**Fig. 1:**
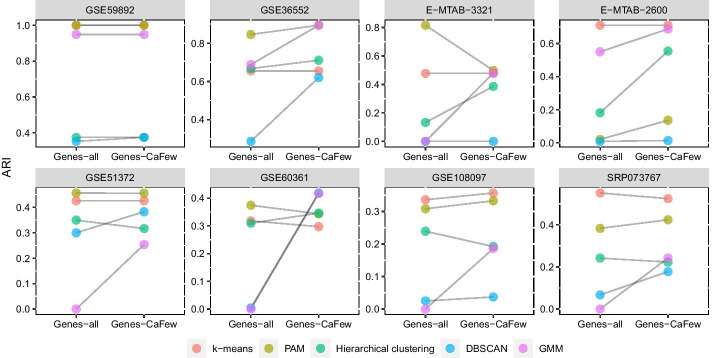
Results of 5 traditional clustering algorithms on 8 scRNA-seq datasets before (*Genes-all*) and after (*Genes-CaFew*) feature selection

We consider five traditional clustering algorithms, including *k-means* [40], *PAM* [41], *DBSCAN* [24], *Hierarchical Clustering* [42] and *Gaussian mixture models* [43]. After feature selection, these algorithms are applied to clustering the 8 datasets. Performance results are shown in Fig. 1.

As shown in Fig. 1, most methods can get improved accuracy on some scRNA-seq datasets after using CaFew to select genes. Concretely, take dataset GSE59892 for example, four algorithms (*k*-means, PAM, Hierarchical Clustering and GMM) maintain their clustering accuracy after feature selection, while DBSCAN is improved. Especially, all methods’ clustering accuracy is significantly improved on two datasets: GSE36552 and E-MTAB-2600. The reason is that CaFew is able to remove some noise genes. However, some methods get degraded clustering accuracy on some datasets after feature selection. This is because that some datasets own relatively complex cluster structures, and the traditional clustering algorithms cannot capture these structures when only hundreds of features are used.

#### Results of clustering methods specifically for scRNA-seq data

**Fig. 2:**
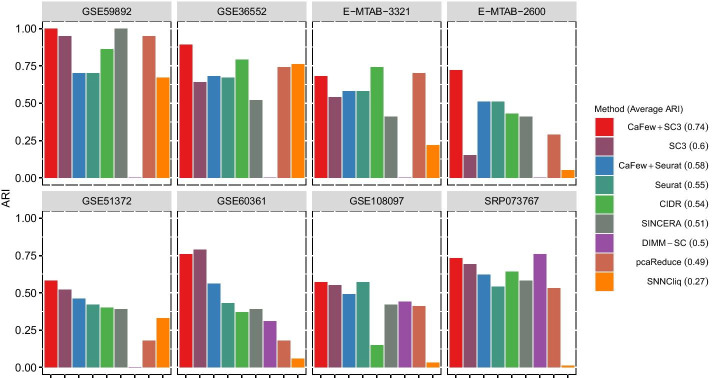
Results of clustering methods specifically for scRNA-seq data. The value in the parentheses following each method’s name in the legend is the average ARI

Based on the previous comparative studies on clustering algorithms for scRNA-seq data [44, 45], we choose several representative methods to test the performance of CaFew. Since SC3 and Seurat achieve the state of the art clustering performance, we mainly test the effect of CaFew on them, and we call them “CaFew+SC3” and “CaFew +Seurat” after using CaFew for gene selection. The results are presented in Fig. 2, where ARI values of different methods are represented by different color bars.

From Fig. 2, we can see that “CaFew+SC3” achieves better performance than other methods on most of the datasets. Its average ARI on the 8 datasets is 0.74, higher than that of the other methods. Concretely, SC3 gets an ARI of 0.94 on dataset GSE59892, while “CaFew+SC3” improves the ARI to 1. For the dataset GSE36552,“CaFew+SC3” performs best with an ARI of 0.89, which is higher than the AIR (0.64) of SC3 and the other six methods. Moreover, the dataset with the largest improvement on clustering accuracy is E-MTAB-2600, and the AIR is improved from 0.15 to 0.72. In general, except for GSE60361, the clustering accuracy of “CaFew+SC3” on the other datasets is better than that of SC3.

As for “CaFew +Seurat”, its clustering accuracy on three scRNA-seq datasets is the same as that of Seurat, gets higher ARI values on four datasets and a lower ARI on only one dataset. We notice that the improvement on ARI for Seurat is not as significant as that for SC3, this is because that CaFew adopts a distance based clustering mechanism like *k*-means, so it is more beneficial to *distance*-based clustering methods such as SC3.

### Visualization

**Fig. 3:**
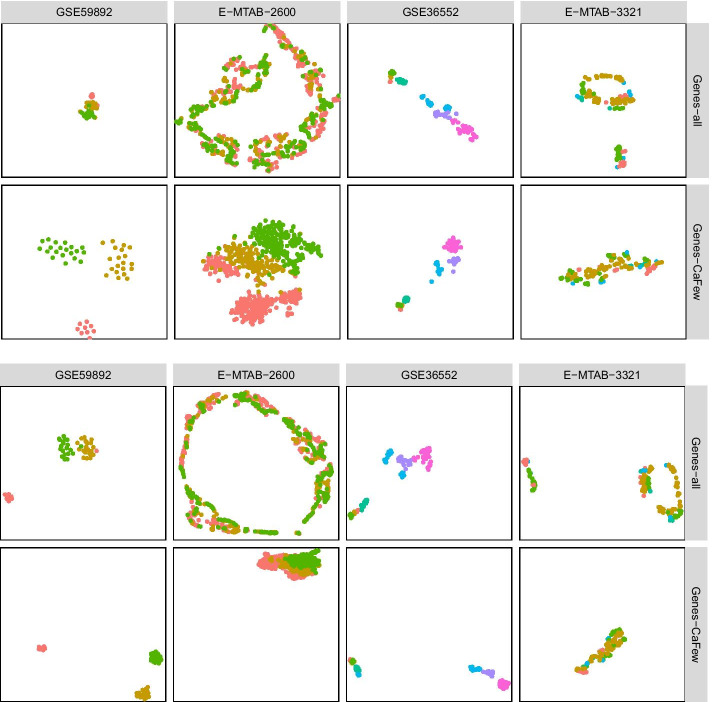
Two-dim visualization results of 4 scRNA-seq datasets before (*Genes-all*) and after (*Genes-CaFew*) feature selection. **a** t-SNE visualization, **b** UMAP visualization

Here we investigate whether CaFew can help with visualization. In our experiments, we adopt t-SNE [46] and UMAP [47] for scRNA-seq data visualization. Figure 3 displays the visualization results of four datasets before and after feature selection. Here, points of similar color belong to the same cluster.

From Fig. 3, we can see that after selecting genes by CaFew, both t-SNE and UMAP can separate different clusters more apart, while making cells from the same cluster closer. This shows that CaFew is beneficial to clustering.

Take the dataset GSE59892 for example, there are 49 samples in 3 clusters with 25,737 features. When all features are utilized, some green and pink points mix with the orange points. After selecting genes by CaFew, the green, pink and orange points are clearly separated.

## Discussion

It is well known that the clustering performance is heavily impacted by the characteristics of input samples. For example, DBSCAN can find clusters of any shape, while *k*-means assumes that clusters are convex shaped. The distribution of samples in different classes may also impact the clustering performance. If the sample distribution is extremely uneven, it is hard for clustering algorithms to find very small clusters such as rare cell types and tumor cells etc. Since CaFew adopts similar idea of *k*-means in the process of weight matrix calculation, the clustering performance on non-convex/uneven distribution data cannot get so significantly improved as on convex/even distribution data. With CaFew, the clustering performance of distance-based methods like *k*-means and SC3 can be considerably improved, but its effectiveness is not so obvious on the other types of methods like Seurat. Additionally, the pre-defined cluster number also affects clustering result. The number of clusters can be determined with prior knowledge or can be estimated by some specific computational approaches. CaFew does not address this issue, but directly uses the exact number of clusters in optimization.

For future work, on the one hand, we will explore alternative clustering optimization mechanism that is not restrict to distance-based clustering, and develop specific methods to determine the number of clusters in the framework of CaFew. On the other hand, we will try to integrate auxiliary biological information into CaFew, and extend this study to the field of differentially expressed gene analysis, especially the study of disease-specific genes.

## Conclusion

In this paper, we propose a novel algorithm CaFew to select features for scRNA-seq data clustering based on cluster-aware feature weighting. By solving the clustering optimization problem, CaFew first obtains the weight matrix *W* of features with regard to different clusters. Then, it filters out genes with small weight in all clusters or a small weight variation across all clusters. Extensive experiments on 8 real datasets show that selecting features with CaFew can boost clustering performance and the combination of CaFew and SC3 achieves the state of the art performance.

## Methods

**Fig. 4:**
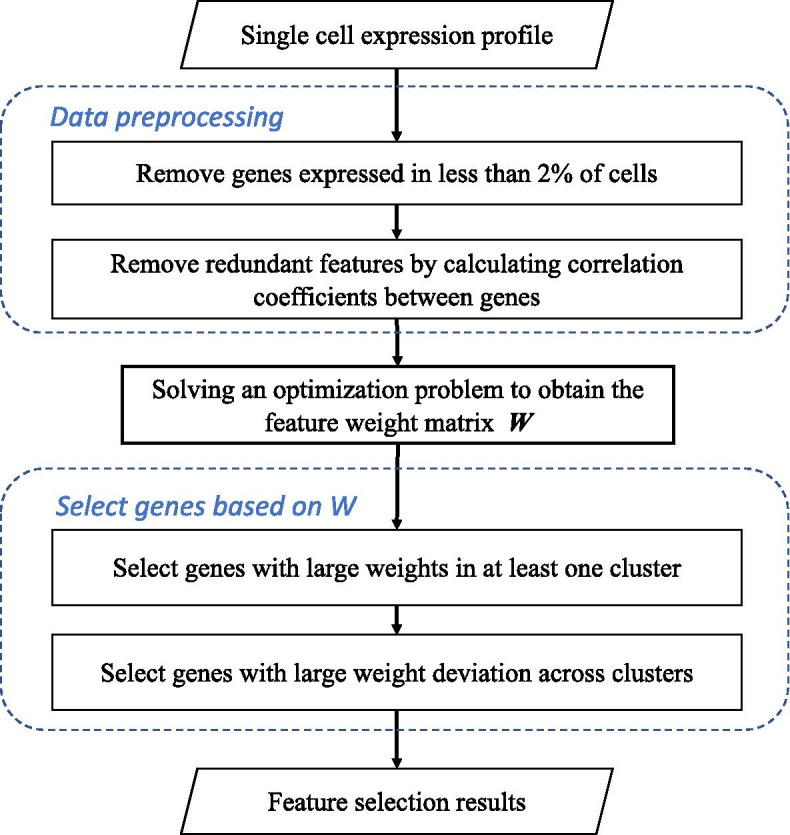
The pipeline of CaFew

In this section, we describe the CaFew method in detail. Figure 4 illustrates the pipeline of CaFew, which consists of three major steps: (1) Removing uninformative and redundant genes; (2) Deriving the feature weight matrix by solving the clustering optimization problem; And (3) selecting genes based on the weight matrix.

### Data preprocessing

In general, scRNA-seq data is extremely sparse, with some genes of zero expression in a large number of cells. The generation of these zero values is due to that these genes are not expressed in these cells, or their genetic products are not detected (also called “dropout events” [17]). Therefore, raw scRNA-seq data are highly uncertain and noisy, which seriously impacts the downstream computational analysis. In the data preprocessing step, we directly delete genes that are expressed only in a small number of cells (less than 2%) [19].

On the other hand, the expression patterns of some genes are very close. If all of them are used for clustering, it only incurs a large amount of calculation cost, but contributes little to clustering. Therefore, we remove the redundant genes. By calculating the Pearson Correlation Coefficient (PCC) between genes, only one gene is conserved as the representative for genes with PCC greater than 0.99.

### Cluster-aware feature weighting

As the importance of each feature (gene) is different across different clusters, we assign different weight to each feature over different clusters, and formulate the clustering objective function as follows [48–50]:

3 $$\begin{aligned} {\begin{matrix} J(K,W;\chi ) = \sum _{k=1}^{K}\sum _{x_{i}\in \chi _{k}}\sum _{j=1}^{n} w_{kj} d_{ki}^{j} +\sum _{k=1}^{K}\delta _{k}\sum _{j=1}^{n} w_{kj}^{2}\\ subject~to,~~w_{kj}\in [0,1]~\forall k,j~and~\sum _{j=1}^{n} w_{kj}=1~\forall k \end{matrix}} \end{aligned}$$

where *K* means the number of clusters, *n* is the number of features (genes), $\chi =\bigcup _{k=1}^{K}\chi _k$ is the union of *K* clusters and $\chi _k$ indicates the *k*th cluster. *W* is a $K*n$ weight matrix and $w_{kj}$ indicates the weight of gene *j* in cluster *k*. A large $w_{kj}$ indicates that feature *j* is important to cluster *k*. $d_{ki}^{j}$ means the distance between sample *i* and the center of cluster *k* on feature *j*. Equation (3) consists of two parts: the first part indicates the sum of distance between samples within each cluster under feature weighting, the second part is the sum of squares of weights, and $\delta _k$ is a parameter to balance the two parts. By minimizing the first part, we can get compact clusters, and by minimizing the second part, we attempt to select as a small number of features as possible for each cluster.

To minimize *J*, according to Lagrange multiplier, we have

4 $$\begin{aligned} {\begin{matrix} J(\Lambda ,W)&= \sum _{k=1}^{K}\sum _{x_{i}\in \chi _{k}}\sum _{j=1}^{n} w_{kj} d_{ki}^{j} +\sum _{k=1}^{K}\delta _{k}\sum _{j=1}^{n} w_{kj}^{2} -\sum _{k=1}^{K}\lambda _{k}(\sum _{j=1}^{n} w_{kj}-1) \end{matrix}} \end{aligned}$$

where $\Lambda = [\lambda _{1},\lambda _{2},...,\lambda _{K}]$ is the Lagrange multipliers. Since the rows of *W* are independent of each other, we can reduce the optimization problem into *K* independent sub-problems. And by setting the derivatives of $J_k$ with respect to $w_{kj}$ and $\lambda _{k}$ to zero, we can derive

5 $$\begin{aligned} w_{kj} = \frac{1}{n} + \frac{1}{2\delta _{k}}\sum _{x_{i}\in \chi _{k}} \left[ \frac{\sum _{j=1}^{n}(d_{ki}^{j})}{n} -d_{ki}^{j}\right] \end{aligned}$$

where the first part $\frac{1}{n}$ is the initial weight (i.e., all features are treated equally), the second part reflects the intra-cluster distance difference between the average over all features and the feature *j*. A positive value of the second part means feature *j* can make the intra-clusters distance smaller and its weight will be become larger than $\frac{1}{n}$. Conversely, a negative value of that part will reduce the weight of feature *j* in cluster *k*.

$\delta _{k}$ is evaluated in an iterative way as follows:

6 $$\begin{aligned} \delta _{k}^{(t)}= C_{\delta } \frac{\sum _{x_{i}\in \chi _{k}^{(t-1)}}\sum _{j=1}^{n}w_{kj}^{(t-1)}(d_{c_{k}i}^{j})^{(t-1)}}{\sum _{j=1}^{n}(w_{kj}^{(t-1)})^{2}} \end{aligned}$$

where $C_{\delta }$ is a constant, the superscripts (*t*) and $(t - 1)$ indicate the current iteration *t* and the previous iteration $(t - 1)$, respectively.

Denote the weighted distance between sample *i* and cluster center *k* as $D_{ik}$, we have

7 $$\begin{aligned} D_{ik}=\sum _{j=1}^{n} w_{kj} d_{ki}^{j}. \end{aligned}$$

Each data point (cell) is assigned to the nearest cluster, that is,

8 $$\begin{aligned} \chi _{k} = \left\{ x_i| D_{ik} \le D_{i{k}'} ~\forall {{k}' \ne k} \right\} . \end{aligned}$$

After the assignment of samples, the cluster centers are updated as follows:

9 $$\begin{aligned} c_{kj}=\left\{ \begin{matrix} 0&{} if &{} w_{kj}=0\\ x_{mj} &{} if &{}w_{kj}\ne 0 \end{matrix}\right. \end{aligned}$$

where $x_{mj}$ is the median value of feature *j* in cluster *k*. By using the median of samples (instead of the mean) to update the cluster center, clustering will be more robust to outliers [51].

Simply, we use Manhattan distance to evaluate $d_{ki}^j$, that is,

10 $$\begin{aligned} d_{ki}^j = \left| x_{ij} - c_{kj}\right| . \end{aligned}$$

### Gene selection based on feature weights

Different from previous feature selection methods, CaFew selects features from the perspective of clusters, instead of cells. On the one hand, feature weight reflects the importance of a feature to a cluster, so the features with large weights are more informative in clustering than those of small weight. On the other hand, a feature whose weight varies greatly among the clusters is usually a “marker” gene that is more conducive to distinguish cells. Based on these two observations, we propose the following strategies to select features.

#### Weight based screening

To remove features with small weight, we first calculate the maximum value of each feature in the weight matrix *W*. Then we divide these features into *N* groups according to their maximum values, where *N*=$3.322*log_{10}(n)-1$, according to the Empirical Sturges’ formula [52]. After arranging the groups of genes in ascending order of their maximum values, we remove the first group of genes, and iteratively use Sturges’ formula to group the remaining features until the number of features is less than 10000. One advantage of this method is that the features of the same interval can be kept as far as possible, instead of some important features being omitted due to the “violent cutting” like simply setting a threshold.

#### Weight-deviation based screening

To further select the “marker” genes for clustering, we measure the variation of feature weights across clusters by *CV* (defined as the ratio of standard deviation over mean). Since there is strong correlation between *mean* and *CV*, we build a linear model to fit *CV* by *mean*: $log(CV^2)=a*log10(mean)+b$ to choose the most significant features of variation. For each feature, we calculate the residual value *d*, which is defined as the difference between the true *CV* and the fitted value. Then, the residual value is normalized as *z*-score: $(d-\overline{d})/\delta$, where $\overline{d}$ and $\delta$ are the mean and the standard deviation of *d*. Finally, *z*-scores are converted into *p*-values with the assumption that all *z*-scores follow normal distribution. In our experiments, we select the features (genes) whose $p \le 0.05$.

### The CaFew algorithm

The pseudo-code of CaFew is outlined in Algorithm 1. Lines 1–3 are for data preprocessing. Line 4 initializes the variables; Lines 5–12 are for deriving the weight matrix of genes; Lines 13–15 are for weight-based gene screening, which removes the genes of small weight; Lines 16-19 are for weight-deviation based screening, which filters genes with small weight variations across clusters.
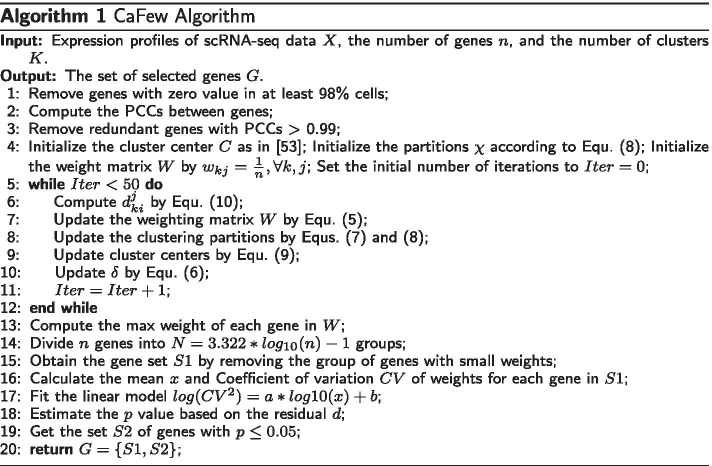


## Data Availability

The datasets used and/or analysed in this study are available from the corresponding articles. Five datasets are available in the GEO repository with accession number GSE59892, GSE36552, GSE51372, GSE108097 and GSE60361 (https://www.ncbi.nlm.nih.gov/geo/query/acc.cgi?acc=GSE59892, https://www.ncbi.nlm.nih.gov/geo/query/acc.cgi?acc=GSE36552, https://www.ncbi.nlm.nih.gov/geo/query/acc.cgi?acc=GSE51372, https://www.ncbi.nlm.nih.gov/geo/query/acc.cgi?acc=108097), https://www.ncbi.nlm.nih.gov/geo/query/acc.cgi?acc=GSE60361.). Two datasets are available in the ArrayExpress repository with accession number E-MTAB-3321 and E-MTAB-2600 (https://www.ebi.ac.uk/arrayexpress/experiments/E-MTAB-3321/, https://www.ebi.ac.uk/arrayexpress/experiments/E-MTAB-2600/.). The dataset SRP073767 is available at https://support.10xgenomics.com/single-cell-gene-expression/datasets/2.1.0/pbmc4k. The source code of CaFew is available at https://github.com/LiRuiyi-raptor/CaFew_Project.

## Abbreviations

scRNA-seq: The abbreviation of single cell RNA-sequencing
CaFew: Cluster-aware feature weighting
SC3: Single-cell consensus clustering
SINCERA: Single cell RNA-seq profiling analysis
CIDR: Clustering through imputation and dimensionality reduction
RaceID: Rare cell type identification
PCoA: Principle coordinate analysis
DBSCAN: Density-based spatial clustering of applications with noise
SNN: Shared nearest neighbors
DIMM-SC: Dirichlet mixture model for clustering droplet-based single cell transcriptomic data
NMF: Nonnegative matrix factorization
SPIN: sorting points into neighborhoods
DL: Deep learning
DCA: Deep count auto-encoder
scVI: Single-cell variational inference
CV: Coefficient of variation
PCC: Pearson correlation coefficient
FPKM: Fragments per kilobase of exon model per million mapped reads
CPM: Counts of exon model per million mapped reads
UMI: Unique molecule identifier
ARI: Adjusted rand index
DBI: Davies–Bouldin index
PAM: Partitioning around medoids
GMM: Gaussian mixture model
t-SNE: t-Distributed Stochastic Neighbor Embedding
UMAP: Uniform manifold approximation and projection
